# Supplementary material for: Mapping the physiological changes in sleep regulation across infancy and young childhood
Source: PLoS Comput Biol. 2024 Oct 21;20(10):e1012541. doi: 10.1371/journal.pcbi.1012541 (PMC11527290; doi:10.1371/journal.pcbi.1012541)
Supplement: S1 Text — Supplementary 2 –Distribution of cost function value for Infant 1. Supplementary 3 –Comparison of optimal parameter combinations with and without period constraint. Supplementary 4 –Summary of fitted parameters for specific sleep pattern periods. (PDF) [file pcbi.1012541.s001.pdf]

Supplementary Material for *Mapping the physiological changes in sleep regulation across infancy and young childhood*

Lachlan Webb[1,*2], Andrew JK Phillips[3], James A Roberts[1,2]

1) Brain Modelling Group, QIMR Berghofer Medical Research Institute, Herston, Brisbane, Queensland, Australia
2) Faculty of Medicine, University of Queensland, Queensland, Australia
3) Flinders Health and Medical Research Institute (Sleep Health), Flinders University, Bedford Park, South Australia, Australia

Selection of results from grid sweep

For sleep duration (Fig Aa), increasing μ increases total sleep duration per 24 h day (TSD), confirming that μ likely decreases with age after starting at a higher value. In comparison, χ has a relatively weak influence on TSD. For number of bouts per day (BPD) (Fig Ab), the dominant parameter is χ, though decreasing μ can also consolidate sleep into fewer bouts. Our fitting results suggest that χ likely increases rapidly to close to adult values, before small changes in χ and the changes in μ determine the timing of the final consolidation to single bout sleep. We found that at higher values of χ there is a compensatory relationship between χ and ν_{vc} (Fig Ac), where consolidation into lower BPD can be caused by either an increase in χ or decrease in ν_{vc}, and a change in one can be offset by a change in other to keep BPD unchanged.

The trajectories of best-fitting parameter combinations (Figs 2 and 3) also indicated that infants have a phase-delayed response to the solar curve compared to adults, with values of b of 0.9-1.0, compared to the typical adult 0.4. Hence, we present firstly Fig Ad with $b = 1.0$, and then Fig Ae with $b = 0.4$.

A newborn with high TSD and BPD will likely therefore have $\nu_{vc} \approx 0$ and $b \approx 1$ with lower $\ln \chi$ and higher μ (Fig Ad, point 1), rapidly over infancy lower in μ and increase in χ as well as develop a circadian rhythm on sleep (Fig Ad, point 2), and over childhood and adolescence move towards the adult value with $\nu_{vc} \approx -3$ mV and $b \approx 0.4$ (Fig Ae, point 3) with $\ln \chi \approx 3.8$ and $\mu \approx 4.2$ nM s.

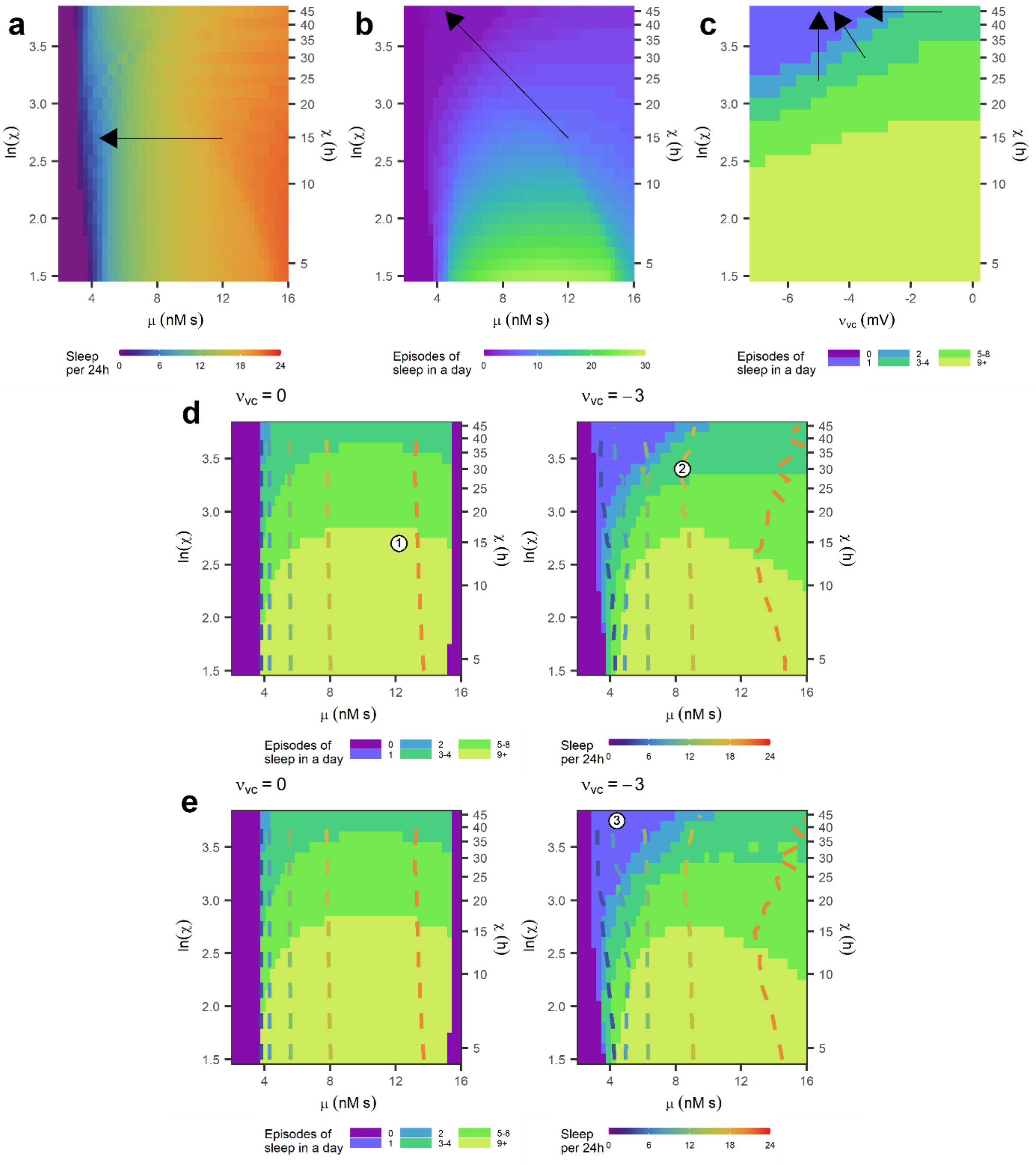

Fig A: Effects of sleep regulation parameters on sleep characteristics. ***a)*** *Total sleep duration (TSD) as a function of μ and $ln(\chi)$. The reduction in TSD with age in infancy and early childhood likely indicates that μ decreases with age (black arrow).* ***b)*** *Number of bouts per day (BPD) as a function of μ and $ln(\chi)$. Consolidation of sleep bouts likely requires χ to increase and μ to decrease (black arrow)* ***c)*** *Bouts per day as a function of ν_{vc} and $ln(\chi)$. At lower numbers of BPD (here for $\mu = 8.1$ nM s), changes in both χ (increases) and ν_{vc} (decreases) can consolidate bouts (black arrows).* ***d)*** *BPD (shading) and TSD (dashed contours) for $b = 1$ and two values of ν_{vc}, Contour lines show 4, 8, 12, 16, and 20 hours of TSD.* ***e)*** *As for d but for $b = 0.4$. Over childhood, an infant would have to move from newborn behaviour of high TSD and many bouts with low ν_{vc} and $b = 1$ (circle 1), to not completely consolidated and still high TSD (circle 2), to adult values (circle 3).*

Distribution of cost function value for Infant 1

Fig B presents the distribution of cost function values (Equation 13) from all parameter combinations considered (Table 2) for the seven-day averaged window of sleep/wake behaviour centred on days 5, 100, 200, 300, 400, and 500 from Infant 1. The high density at the low end of the distribution for Day 5 shows the lack of identifiability in the results, where many parameters can produce sleep/wake behaviour with a similar cost function value. By contrast, for the later days (300, 400, and 500) the lower tails are flat, indicating that only a small number of parameter combinations can reproduce similar sleep/wake patterns.

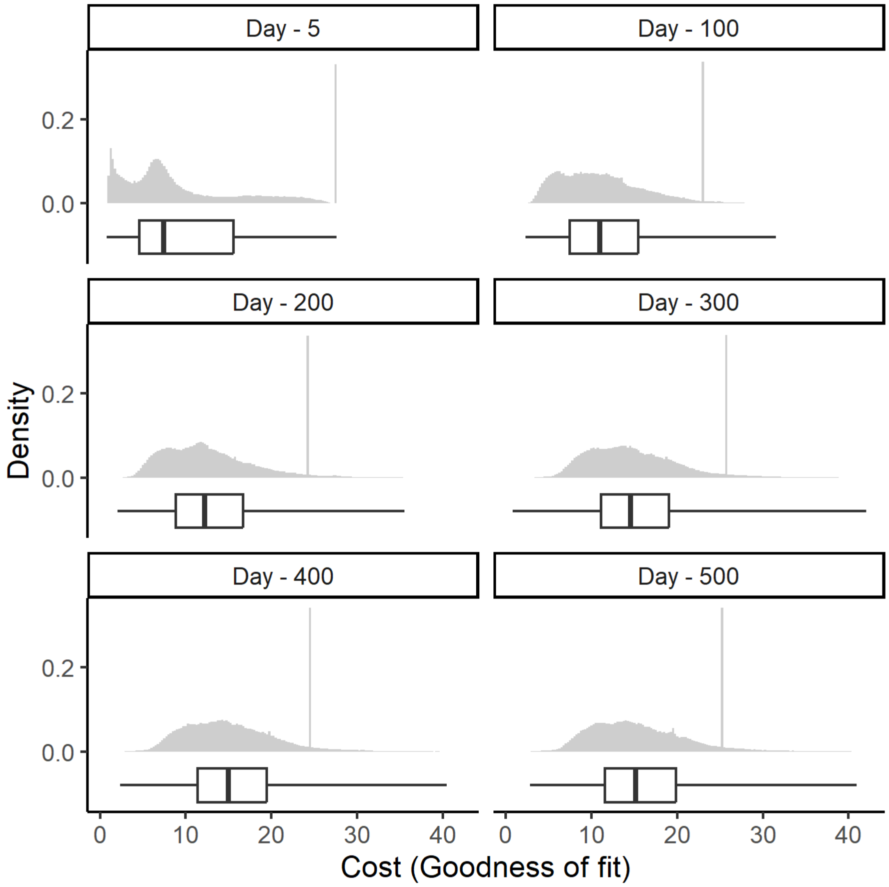

__Fig B:__ The distribution of cost function values for specific times in Infant 1 across the entire set of explored parameter combinations. The sharp spikes in density arise from the multiple combinations in the parameter space that produce permanent sleep. Boxplots indicate the median and upper and lower quartiles, with whiskers extending to the maximum and minimum.

Comparison of optimal parameter combinations with and without period constraint

Fig C presents the effect of removing the pacemaker period constraint when identifying the optimal parameter combination when fitting to Infant 1.

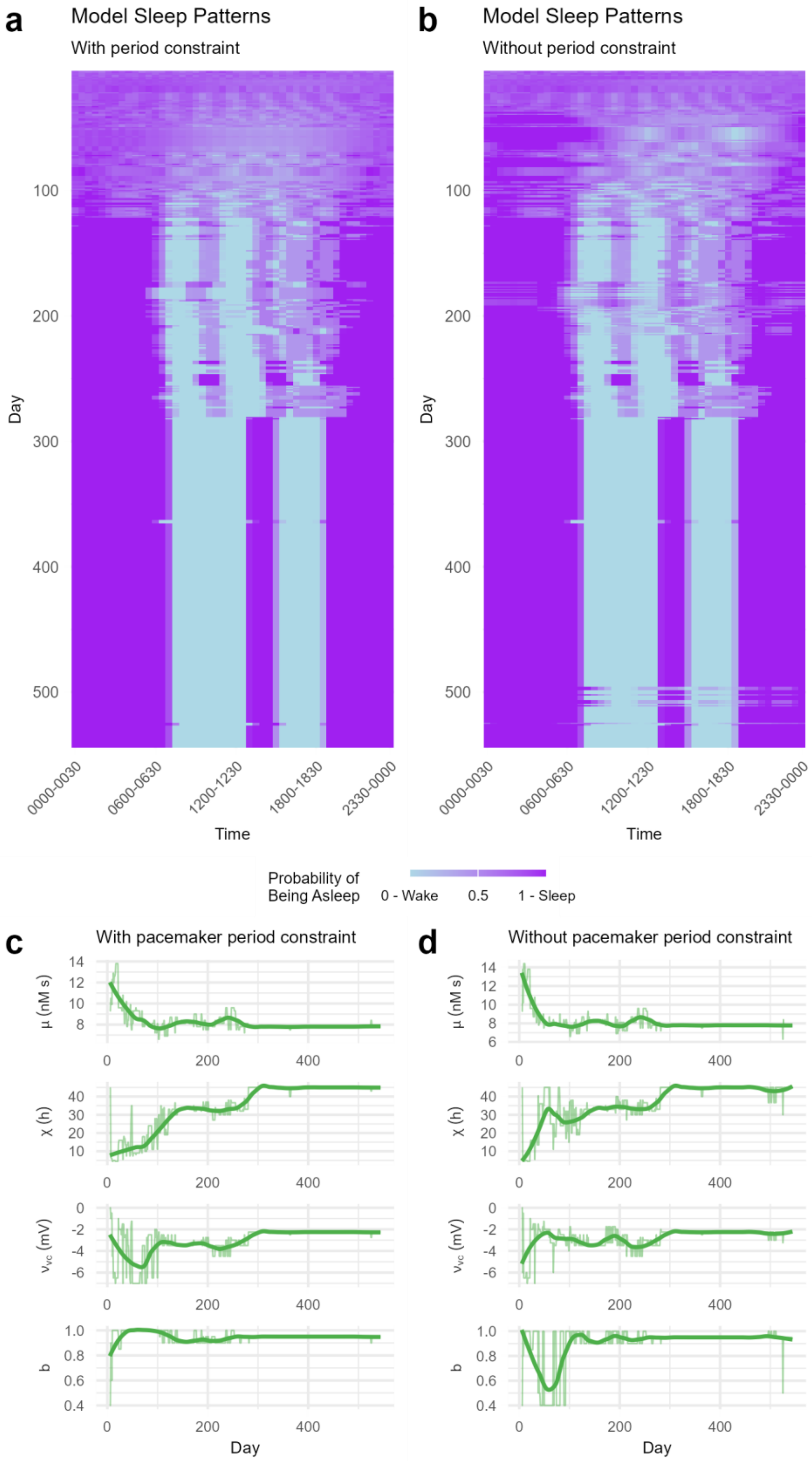

Fig C: Fitted trajectory of sleep maturation for Infant 1, with and without pacemaker period constraint. a) The sleep/wake patterns for each best-fitting parameter combination, replicated from Fig 2b for visual comparison. b) The sleep/wake patterns for each best-fitting parameter combination, fitted without the pacemaker period constraint. c) The trajectories of best-fitting parameter combinations, superimposed with a Loess smoothed line (thick lines) with smoothing parameter $\alpha_{Loess} = 0.2$ that produce the sleep/wake patterns in a), replicated from Fig 2c for visual comparison. d) The trajectories of best-fitting parameter combinations, superimposed with a Loess smoothed line (thick lines) with smoothing parameter $\alpha_{Loess} = 0.2$ that produce the sleep/wake patterns in b).

Summary of fitted parameters for specific sleep pattern periods

Days were manually chosen from inspecting the original data. Best fit model parameters from time windows bound by those days were then summarised.

***Table A**: Mean (SD) fitted parameter values in 1- and 2-nap sleep patterns.*

Sleep patterns	Infant 1	Infant 2	Infant 3
2 naps	Day 110 - 275	Day 290 - 510	Day 110 - 345
μ nM s^{-1}	8.20 (0.57)	7.75 (0.51)	5.86 (0.53)
χ h	32.4 (3.55)	30.6 (1.99)	32.0 (5.51)
ν_{vc} mV	-3.45 (0.44)	-3.73 (0.63)	-1.62 (0.53)
b	0.93 (0.04)	0.97 (0.02)	0.989 (0.03)
1 nap	Day 280 - 548	Day 520 - 535	Day 350 - 370
μ nM s^{-1}	7.80 (0.06)	7.8 (0)	5.82 (0.25)
χ h	44.9 (0.56)	41.0 (5.18)	36.6 (1.55)
ν_{vc} mV	-2.26 (0.05)	-2.55 (0.39)	-1.28 (0.13)
b	0.95 (0.004)	0.93 (0.03)	0.99 (0.05)
