# Supplementary material for: Mapping the physiological changes in sleep regulation across infancy and young childhood
Source: PLoS Comput Biol. 2024 Oct 21;20(10):e1012541. doi: 10.1371/journal.pcbi.1012541 (PMC11527290; doi:10.1371/journal.pcbi.1012541)
Supplement: Attachment — Submitted filename: PCOMPBIOL-D-24-00193.pdf [file pcbi.1012541.s002.pdf]

The authors explored the applicability of an established model for sleep regulation in adults to model infant sleep, and examined the necessary adjustments in key parameters related to sleep homeostasis and circadian rhythms to accommodate developmental changes. They utilized longitudinal data from four infants for their investigation. This approach is both intriguing and crucial, representing the initial endeavor to model infant sleep patterns. However, significant clarifications are required, as outlined in the comments below.

Results:
There is probably some noise in the observed sleep/wake patterns as common in biological data. What role could noise play in your model?
Fig. 3 shows a very stable pattern of the probability to be asleep or awake in the second half of the model sleep patterns. Further, the two wake bouts are not quite at the correct phase and the afternoon wake bout is shorter than the empirical one.

The smoothed trajectories of the best-fitting parameter combinations don't show a monotonic time course as one might expect. What is your interpretation?

Looking at the patterns in Fig. 2, in dataset 1 to 3 you can identify roughly three pattern over time: irregular sleep/wake, rhythms emerging; two daily naps; one nap per day. The timing of the naps and the abrupt switch from two naps to one nap is probably not mainly driven by biology but rather a decision of the mother/parents. How does such a decision of the parents relate to a change in the estimated parameters? Please discuss and elaborate on it.
How about having distinct fixed parameters (estimated for the corresponding time period) for the different patterns occurring over time?

"All four datasets appear to converge to individual-specific values of the parameters, ..." How do you define converge? It does not seem to apply to all parameters.

When fixing some parameters what is the rational for using the particular values?

Fig. 5: also provide the parameter values when you fixed two of them.

Fig. 5 b,c: how was "Total Cost" determined?

Did you run simulations with parameters corresponding to the values of the smoothed parameter curves? Would be very interesting to see the resulting patterns.

Discussion:
"The model could also be used to develop optimised sleep schedules for children, ..." why should sleep schedules be optimized in infants?

Jenni et al. (2005) investigated developmental changes in the homeostatic process during adolescence and found that the buildup of the homeostatic process got slower (longer time constants for buildup) with increasing age whereas the dissipation of sleep pressure (time constant of exponential decline) of remined similar. Further, the distance between the asymptotes (upper and lower) was increasing with age. How would these observations relate to your findings at an earlier age?

Methods:
Implementation: Overall, the description lacks adequate detail.

“The model was solved for an initial time of 4 weeks to remove transients caused by initial values, then using the last value of the initial run as the starting values”. Which parameters were used? Does this apply to each point in the grid search?

“..., solved for a time period of 35 days to produce a sleep/wake state time series. We calculated sleep characteristic summary measures on the last 14 days of the generated time series.” Same questions as above.

Parameter grid search: did you explore all possible parameter combinations or how is simultaneous varying of parameters to be understood?

Fitting to empirical data: step size for the sliding window? Was an overlap implemented? Different parameter combinations might lead to “optimal” cost values. How do you deal with such a situation. How does the search relate to Supplementary Fig. 2? This figure is insufficiently described.

Typo:
“Systematically fixing one parameter (cool colours in Figure 6a) or ...” should be Figure 5a.
