# Supplementary material for: Mapping the physiological changes in sleep regulation across infancy and young childhood
Source: PLoS Comput Biol. 2024 Oct 21;20(10):e1012541. doi: 10.1371/journal.pcbi.1012541 (PMC11527290; doi:10.1371/journal.pcbi.1012541)
Supplement: Attachment — Submitted filename: PLOS CB Response to Reviewers 20240703.docx [file pcbi.1012541.s003.docx]

Response to Reviewers for "**Mapping the physiological changes in sleep regulation across infancy and young childhood**" for consideration at **PLOS Computational Biology**.

Reviewer comments are in *italics*, response to reviewers are in blue, and manuscript text is in red with edit to texts **bolded red**. Citations that have added are given in Author (year) format as opposed to the squared numbered referencing system in the manuscript.

We thank the reviewers for their helpful feedback, and appreciate the opportunity to clarify some points and improve our manuscript.

**Reviewer 1**

*Reviewer #1: The manuscript presents a mathematical model attempting to explain developmental changes in infant sleep. Applying this model to longitudinal sleep data from 4 infants confirms the faster accumulation and clearance of sleep homeostatic pressure and the weaker circadian rhythm in this sample as compared to previous observations in adults. Additionally, greater sensitivity to phase-delaying effects of light is observed in the presented dataset. Taken together, this work represents an important step to understanding the mechanisms contributing to the large inter-individual variability in sleep regulation during infancy. The integration of knowledge from different fields including neurobiology and computational modelling to address this complex question is a key strength of the study. However, the limitation stemming from the fact that the four datasets correspond to only four individual subjects should be more explicitly acknowledged in the manuscript, as it dampens overall enthusiasm for the study's findings. Furthermore, the manuscript would benefit from a more nuanced discussion of the contextual factors influencing infant sleep as well as future directions regarding mathematical modeling thereof. Finally, I recommend several minor changes to improve the readability of the manuscript (see below).*

We thank Reviewer 1 for their positive appraisal of our work, and have responded to each of their points below.

*Results
Sleep-wake switch model (page 3): I suggest moving Figure 1 to the Methods section in order to make clear that this is a previously validated model that was used and not developed in the scope of the current study.*

We have moved the previous Figure 1 (now Figure 5) and relevant text to the Methods section (page 15).

*Empirical sleep patterns (page 3): I recommend referring to e.g. “subjects” instead of “datasets” throughout the manuscript to make clear that data from four individuals was used in the study.*

We have changed each usage of “datasets” to “infants” throughout the manuscript.

*The parameter space of sleep maturation (page 5, “…the circadian rhythm in sleep is non-existent in the days soon after birth.”): I strongly recommend rephrasing this sentence to accurately reflect the body of evidence indicating that circadian rhythms begin developing already before birth. The term 'non-existent' is overly strong; although sleep patterns in the first few weeks may not yet resemble those of adults or older children, there is significant variability among infants. In some cases, clear diurnal tendencies can be observed shortly after birth.*

The reviewer raises important points. There is indeed good evidence that the foetal superchiasmatic nucleus produces circadian rhythms in utero, though it is also believed that the foetal SCN receives signals from the maternal circadian system (Wong, et al. 2022). At birth, the infant loses this signal source, and the functional yet immature circadian system must develop. This is seen in not only sleep, but melatonin and cortisol as well (Wong, et al. 2022). While these rhythms can develop quickly (in the order of days to weeks), our statement that “circadian rhythm in sleep is non-existent in the days soon after birth” was in reference to the days immediately after birth as the infant changes from the maternal circadian rhythm to their own developing rhythm, which is particularly relevant for Infants 1 and 4 as the data begins in the days after birth. We have changed this sentence to accurately reflect the point we were trying to make:

Page 5

Changes in 𝜈_𝑣𝑐_ have already been suggested in the literature [30-32], and in particular McGraw, Hoffmann, Harker and Herman [25] and Jenni, Deboer and Achermann [24] showed that the circadian **influence on** **sleep tends to be close to zero in the days soon after birth as the infant loses the mother’s circadian signal and their own circadian system (already active in utero (Wong, et al. 2022, Serón-Ferré, et al. 2012)) matures in response to the ex utero environment**.

*Trajectories through parameter space inferred from empirical data (page 5-6): I believe this section would benefit from a more precise and quantifiable presentation of the results, rather than the current descriptive approach. For example, in the last sentence on page 5, the authors mention that the homeostatic clearance time “starts small at around 5-10 h for some time”, which is very vague.*

We have made our descriptions more precise:

Pages 5-6

The trajectories of each parameter in the best-fitting combinations (Figure **2**c), although noisy, exhibit clear trends. For example, 𝜇 (the rise in homeostatic pressure from wake) is high in the early months of **Infant** 1, **reaching a maximum of 13.8 nM s^-1^ for the windows centred on days 17 to 21**, before settling around 𝜇=8 nM s^−1^ for the rest of the trajectory **(mean (SD) after timepoint centred on Day 120 of 𝜇=7.97 (0.388))**. The circadian strength parameter 𝜈_𝑣𝑐_ starts close at zero (aligning with prior observation that there is negligible circadian influence on sleep-wake cycling at birth [24, 25]), before increasing in strength to between **-2.25 to -4.5** mV after day 100. The homeostatic clearance time, 𝜒, starts small at around 5-10 h for some time **(smoothed 𝜒 estimate below 10 h until timepoint centred on Day 30)**, then increasing over time to eventually become relatively constant **at between 30 and 35 h from 130 day to 265 days**, then increases again to **45 h from 280 days onwards**. The parameter 𝑏 is high (𝑏>0.95, versus adult 𝑏=0.4) and shows a slight decrease over time.

*Trends of best-fitting parameter combinations for all datasets (page 7): I appreciate that parental interventions are acknowledged as a potential confounding factor. However, I believe that including further information about what these parental interventions included, and under which circumstances the data were collected is important for the interpretation of the current findings. I understand that this information is provided elsewhere, however, given its relevance to the current study, I believe it should be directly accessible without the need to consult other publications.*

We agree with the reviewer’s point and have added more details about the datasets into this section, in particular Infant 4:

Page 7

For **Infants** 1 and 3, we found an early rapid decrease in 𝜇, while **Infant** 2 starts much later (3 months) so appears to have missed that initial decrease. **Infant** 4 has a slower decrease in 𝜇, possibly due to the lack of parental intervention in sleep patterns [25] as the **parents of Infant 4 explicitly did not influence the sleep patterns of the infant**. **Hence the slower decrease in 𝜇 in Infant 4 may be a more natural decrease, compared to the faster decreases with the other infants possibly being influenced by parental sleep schedule decisions.**

…

Page 8

The strong 𝜈_𝑣𝑐_ values at the beginning of some of the trajectories (i.e., **Infants** 2 and 3) could reflect parental intervention in the timing of sleep bouts throughout the day, rather than the endogenous signal from the SCN. **Infant** 4 (**where the parents aimed to not explicitly influence the sleep patterns of the infant**), began with a small 𝜈_𝑣𝑐_ as the best fit followed by an increase in strength.

*Relative contributions of parameters at different ages (page 9): I believe the figure reference should read “Figure 5a” instead of “Figure 6a”.*

We thank the reviewer for alerting us to this typo, we have corrected it.

*Discussion
Page 10 (“…sleep homeostatic clearance rapidly slowing, and sleep homeostatic accumulation…”): I believe sleep homeostatic clearance and accumulation should be reversed in the last sentence, particularly given that the findings regarding clearance are not as conclusive.*

We have rearranged the sentence to the following:

Page 11

We found that the underlying biological processes appear to mature at different rates, with the strength of circadian rhythms increasing rapidly after birth**,** **sleep homeostatic accumulation and the circadian system’s response to light maturing slowly, with these processes likely continuing into childhood or adolescence, and sleep homeostatic clearance rapidly slowing over infancy**.

*Page 11: In the last paragraph before the limitations, the sentence “…it is
possible that both developmental changes in both…” is not clear. I suggest rephrasing. The same paragraph would benefit from a more direct discussion of the implications regarding the relationship between the two parameters. Could the authors suggest alternative modeling approaches to overcome this issue? What would these “further constraints” encompass?*

We have restructured the paragraph as follows to make our point clearer:

Page 12

**Newborn and early infant sleep is characterized by high BPD, with many night awakenings and bouts of sleep throughout the day. Both fast homeostatic clearance (low 𝜒) and weak circadian drive (low |𝜈𝑣𝑐|) can produce polyphasic sleep (BPD > 1), though to produce BPD > 4 a lower 𝜒 is required.** While it is expected that the circadian drive to sleep would start at close to 0 before strengthening [24, 25], **our modelling suggests that both 𝜒 and 𝜈𝑣𝑐 have different values in infancy versus adulthood, and thus developmental changes in both parameters would occur during infancy.**

We have also added the following to address the questions regarding modelling approaches and constraints at the end of the paragraph in question.

Page 13

… without a further constraint. **Such a constraint could be informed by animal studies into the development of the sleep homeostatic process in infancy (e.g. sleep deprivation studies), or modelling circadian markers such as cortisol or core body temperature.**

*Page 12: The manuscript mentions the role of parental interventions and external environmental factors but does not provide a detailed discussion of how these might affect the model's accuracy or the interpretation of results. I recommend incorporating a more explicit elaboration on the implications of such factors for the current study’s findings as well as for similar modeling approaches in the future.*

We have expanded upon this point and added the following:

Page 13

Moreover, interventions by parents/carers can change an infant’s sleep schedule, for example by encouraging naps at certain times, attempting to consolidate naps, or promoting sleep onset at night. **While we expect development is a continuous process, there exists in the model parameter space between the 2-nap and 1-nap sleep behaviour, combinations of parameters that produce behaviour which alternates between 1-nap and 2-naps. Parents may intervene at this point to impose a 1-nap solution. These external forces may result in changes in parameters that do not actually reflect changes in the underlying physiological processes, thus reducing the model’s accuracy and affecting the interpretation**. Notably, this model can be used to simulate constraints on sleep timing [16, 17, 41], meaning that such behaviours could theoretically be incorporated to more accurately model individual circumstances. Future work could also incorporate effects of other environmental stimuli, such as noise [20] **or sleep-encouraging behaviours from parents, to more accurately describe the inputs to the sleep model.**

*Similarly, while the last paragraph notes the clinical relevance of such modeling approaches, the description of potential future directions remains superficial. I believe the conclusion would benefit from more specific suggestions for future applications.*

We have expanded upon this point and added the following:

Page 14

Beyond understanding natural development, our modelling framework could also be used to understand the underpinnings of sleep disorders in children, for example by inferring the model parameters for specific disorders**, e.g. sleep disordered breathing (Menzies et al. 2022).** **This could lead to an improved understanding of the underlying neurobiology of such disorders, as well as revealing model informed ways to address these issues.** The model could also be used to develop optimised sleep schedules for children, **where improvements in sleep could lead to improvements in neurodevelopment and cognition,** similar to work that has been done for adolescence [16, 43]. We thus anticipate that sleep modelling will be a valuable tool in paediatric sleep research.

*Methods
Overall, I believe this section could be structured more clearly. For example, on several occasions, the variables used in the equations are introduced rather late. For example, the reader must wait until equation 4 to understand v which already appears in equation 1.*

We have rearranged the Methods section so it can be more easily followed. The sentence describing the $\nu_{ab}$ term is now prior to the paragraph describing the sleep drive. It now reads as:

Page 15

The mean firing rate and mean cell body potential of the neuron populations $j=m,v$are$Q_{j}$ and$V_{j}$, respectively, and 𝐴 is excitatory input from the orexinergic and cholinergic neurons **(Phillips and Robinson 2007; Phillips and Robinson 2008)**. **The** $\boldsymbol{\nu}_{\boldsymbol{ab}}$**terms represent the strength of the input/connection from the neuron population/drive 𝑏 into neuron population 𝑎. For example,** $\boldsymbol{\nu}_{\boldsymbol{mv}}$ **is the strength of the VLPO input into the MA group.**

*Furthermore, referencing previous work, particularly on page 13 would help the reader understand how established which parts of the modeling process are and how exactly the current study builds on this work to adapt the approach to the new application.*

We have added references throughout this section to assist the reader. While we have used a numbered reference system in the manuscript, for readability of this response in the text below we indicated the author and year:

Page 15-16

The mutual inhibition between the MA and VLPO neuron populations is governed by Equations 1 and 2 **(Phillips and Robinson, 2007)**, respectively,

$$\tau_{m}\frac{dV_{m}}{dt}=-V_{m}-\nu_{mv}Q_{v}+A, (1)$$

$$\tau_{v}\frac{dV_{v}}{dt}=-V_{v}-\nu_{vm}Q_{m}+D, (2)$$

where

$$Q_{j}=\frac{Q_{\max}}{1+exp (-(V_{j}-\theta)/\sigma)} , j=m,v. (3)$$

The mean firing rate and mean cell body potential of the neuron populations $j=m,v$ are $Q_{j}$ and $V_{j}$, respectively, and $A$ is excitatory input from the orexinergic and cholinergic neurons **(Phillips and Robinson 2007; Phillips and Robinson 2008)**. **The** $\boldsymbol{\nu}_{\boldsymbol{ab}}$**terms represent the strength of the input/connection from the neuron population/drive 𝑏 into neuron population 𝑎. For example,** $\boldsymbol{\nu}_{\boldsymbol{mv}}$ **is the strength of the VLPO input into the MA group.**

The sleep drive to the VLPO is the term $D$ in Equation 4, which is the weighted sum of the sleep homeostatic process ($H$), circadian process ($C$), and constant background input to the VLPO ($D_{0}$) which represents the sum of all other inhibitions and excitations to the VLPO **(Fulcher et al., 2008)**:

$$D=\nu_{vh}H+\nu_{vc}C+D_{0}. (4)$$

The sleep homeostatic process $H$, the process by which sleep-inducing chemicals such as adenosine build up in the brain (particularly the basal forebrain) during wakefulness and clear during sleep, obeys

$$\chi\frac{dH}{dt}=-H+\mu Q_{m}, (5)$$

where $\chi$ is the characteristic clearance time of the somnogens, and $\mu$ is the rate of increase in the level of somnogens, assumed to be proportional to the activity of the wake-promoting group MA, due to the correspondence between MA activity and arousal state **(Phillips and Robinson, 2007).**

The circadian process $C$ is defined by

$$C(t)=12(1+0.80y-0.47x),(6)$$

where $x$ and $y$ are the variables of a forced, modified van der Pol oscillator **(Phillips et al., 2010)** defined by

$$\kappa\frac{dx}{dt}=\gamma\left( x-\frac{4x^{3}}{3} \right)-y\left( \left( \frac{24}{f\tau_{c}} \right)^{2}+kB \right), \left( 7 \right)$$

$$\kappa\frac{dy}{dt}=x+B, (8)$$

$$\frac{dn}{dt}=\lambda\left( \alpha_{0}\left( \frac{\tilde{I}}{I_{0}} \right)^{p}\left( 1-n \right)-\beta n \right), (9)$$

where n is the fraction of currently ready photoreceptors and B is the drive from the photoreceptive pathway to the circadian pacemaker **(Forger et al., 1999)**,

$$B=G\left( 1-n \right)\left( 1-bx \right)\left( 1-by \right)\alpha_{0}\left( \frac{\tilde{I}}{I_{0}} \right)^{p}, \left( 10 \right)$$

$$Ĩ=\left\{ \begin{aligned} \begin{matrix} L\left( t \right), & V_{m}>V_{v} \end{matrix} \\ \begin{matrix} 0, & V_{m}\leq V_{v} \end{matrix} \end{aligned} \right., (11)$$

and where $L(t)$ defines the light level, with

$$L(t)=l_{e}+\frac{\left( l_{d}-l_{e} \right)}{2}\tanh\left( c\left( t-s_{1} \right) \right)-\tanh(s(t-s_{2})). (12)$$

*Extracting and converting the empirical data (page 16): I strongly recommend describing the four datasets in more detail. For instance, I believe the sex of the four infants is not mentioned in the manuscript. Additionally, the fact that the four datasets employ different methods for sleep data collection (sleep diary vs. actigraphy) should be acknowledged as a limitation, and their comparability should be further discussed. Furthermore, the difficulty of deriving reliable sleep data from infant actigraphy should be acknowledged (e.g., Schoch et al., 2019, SLEEP). Lastly, the methodology for deriving sleep data from dataset 4 is unclear. Could the authors provide more information, e.g., about the specific cut-off mentioned?*

We thank the reviewer for their many suggestions, which we address in turn.

Unfortunately, the sex of the infants is not available for all. However, we have included the sex information on page 18:

**Infant** 1:

Sleep diary data taken from the Agenoria package (https://github.com/jiuguangw/Agenoria) collected **from a male infant** by Jiuguang Wang (www.robo.guru), used for academic purposes under CC BY-NC-SA 4.0. Data was recorded as sleep onset and offset time. We used only complete days of data.

**Infant** 2:

Sleep diary data taken from the Baby-data-viz package (jitney86 https://github.com/jitney86/Baby-data-viz), used for academic purposes under MIT license. Data was recorded as whether the infant was awake, awake and feeding, or asleep, in 15 minute intervals. We used only complete days of data. **No information on biological sex given.**

**Infant** 3:

Actigraphy data **from a female infant**, converted to rest/activity using a cut-off of 5 activity counts per minute as per Jenni, Deboer and Achermann [24]. Periods of one hour or more with zero activity were considered missing data and removed.

**Infant** 4:

Sleep diary data taken from a scan of the published image from McGraw, Hoffmann, Harker and Herman [25]. Data **from a male infant** were originally manually recorded, and were published with feed times, sunsets, sunrises and other information overlaid.

The different data collection methods were already noted as a limitation in the Discussion, but we have added the below sentence to the Methods to make it clearer.

Page 19

**It should be noted that the data collection method for each subject is different, which is a clear limitation to this work.**

The acknowledgement of the difficulty of reliably estimating sleep data from actigraphy has been extended, and also included the reference the reviewer suggested.

In the Discussion on page 13:

Second, while we found differences in the parameter trajectories between the **infants**, the limited sample size makes it difficult to draw conclusions about the heterogeneity in sleep patterns in infancy, as well as the differences in recording methods (sleep diary vs actigraphy) which can bias estimates in TSD and sleep onset time [42] **with the particular complications of using actigraphy in infancy well documented (Schoch, et al. 2022) including underestimating day time sleep and dependence on classification algorithm. Heterogeneity in recording methods likely reduces the identifiability of parameters at the cohort level; future work would benefit from large datasets with fixed recording protocols.**

We have added more details on how we derived the data from Infant 4 on page 18:

Infant 4:

Sleep diary data taken from a scan of the published image from McGraw, Hoffmann, Harker and Herman [25]. Data **from a male infant** were originally manually recorded, and were published with feed times, sunsets, sunrises and other information overlaid. The image was scanned in black and white**, imported to Matlab as numeric array via the imread function (scale from 0 to 255 where black is 0 and white is 255)**, and a cut-off used to distinguish between wake and sleep **(cut-off of 200)**. Horizontal lines of pixels were manually chosen for extraction to represent each day. Extra information (sunrise, sunsets etc.) was removed manually.

*Fitting to Empirical Data (page 16): The 23.995 to 24.005-h period in the last sentence seems narrow. Can the authors justify this choice and have they tested other ranges?*

This is an interesting point and we thank the reviewer for raising it. The primary reason for using this constraint was to correctly identify cases where the system was entrained, generating a daily rhythm with a 24-hour observed period. A narrow range is required to avoid misclassifying non-entrained patterns with a period close to but not equal to 24 hours. We have now added a supplementary figure showing the effect of removing the 23.995-24.005 period constraint (Supplementary 3).

**Supplementary Figure 3 presents the effect of removing the pacemaker period constraint when identifying the optimal parameter combination when fitting to Infant 1.**

**Supplementary Figure 3:** Fitted trajectory of sleep maturation for Infant 1, with and without pacemaker period constraint. a) The sleep/wake patterns for each best-fitting parameter combination, as in Figure 3.5. b) The sleep/wake patterns for each best-fitting parameter combination, fitted without the pacemaker period constraint. c) The trajectories of best-fitting parameter combinations, superimposed with a Loess smoothed line (thick lines) with smoothing parameter $a_{Loess} = 0.2$ that produce the sleep/wake patterns in a). d) The trajectories of best-fitting parameter combinations, superimposed with a Loess smoothed line (thick lines) with smoothing parameter $a_{Loess} = 0.2$ that produce the sleep/wake patterns in b).

**Reviewer 2**

*The authors explored the applicability of an established model for sleep regulation in adults to model infant sleep, and examined the necessary adjustments in key parameters related to sleep homeostasis and circadian rhythms to accommodate developmental changes. They utilized longitudinal data from four infants for their investigation. This approach is both intriguing and crucial, representing the initial endeavor to model infant sleep patterns. However, significant clarifications are required, as outlined in the comments below.*

We thank the reviewer for the positive appraisal of our work.

*Results:*

*There is probably some noise in the observed sleep/wake patterns as common in biological data. What role could noise play in your model?*

We agree that the biological data these results are based on likely contain noise. While we have limited our work to a deterministic model there would be scope in future to consider a sleep model with noise included. We have added the following the Discussion on page 13:

Nevertheless, we expect that the longer and slower changes do reflect the main features of sleep maturation. **We anticipate that incorporating noise in the model would lead to greater day-to-day variability in the sleep/wake patterns, and potentially a greater number of sleep bouts. This may change some of the parameter estimates.**

*Fig. 3 shows a very stable pattern of the probability to be asleep or awake in the second half of the model sleep patterns. Further, the two wake bouts are not quite at the correct phase and the afternoon wake bout is shorter than the empirical one.*

We have added commentary on this observation on page 5.

… and persistence of this regime up to around day 280 where the sleep pattern evolves from two naps to a single nap each day. **While the single nap sleep pattern is consistent throughout the second half of model fit data, the timings of waking and falling asleep do not completely align with the data.** At the start of the trajectory (up to day ∼100) there is large variability in 𝜒 and 𝜈_𝑣𝑐_, with these parameters …

*The smoothed trajectories of the best-fitting parameter combinations don’t show a monotonic time course as one might expect. What is your interpretation?*

This is an excellent question. We have added an acknowledgement of this in the Results section on page 6:

…parameter 𝑏 is high (𝑏>0.95, versus adult 𝑏=0.4) and shows a slight decrease over time. **While there are clear trends, there also exists evidence of non-monotonicity in how the parameters change with development.**

And commentary in a paragraph in the Discussion on page 13:

Hence some sudden jumps in inferred parameter values may reflect the model attempting to compensate for external influences. **The observed non-monotonicity in the trends may also be a result of these external forces. Other causes may be identifiability issues, as well as the interdependence between parameters leading to any non-monotonicity in one parameter being reflected in other parameters.** Nevertheless, we expect that the longer and slower changes do reflect the main features of sleep maturation.

*Looking at the patterns in Fig. 2, in dataset 1 to 3 you can identify roughly three pattern over time: irregular sleep/wake, rhythms emerging; two daily naps; one nap per day. The timing of the naps and the abrupt switch from two naps to one nap is probably not mainly driven by biology but rather a decision of the mother/parents. How does such a decision of the parents relate to a change in the estimated parameters? Please discuss and elaborate on it.*

It would potentially be impacted by it. We have added commentary to the Discussion regarding this point on page 13:

Moreover, interventions by parents/carers can change an infant’s sleep schedule, for example by encouraging naps at certain times, attempting to consolidate naps, or promoting sleep onset at night. **While we expect development is a continuous process, there exists in the model parameter space between the 2-nap and 1-nap sleep behaviour, combinations of parameters that produce behaviour which alternates between 1-nap and 2-naps. Parents may intervene at this point to impose a 1-nap solution. These external forces may result in changes in parameters that do not actually reflect changes in the underlying physiological processes, thus reducing the model’s accuracy and affecting the interpretation**.

*How about having distinct fixed parameters (estimated for the corresponding time period) for the different patterns occurring over time?*

We would expect development processes reflected by some level of continual evolution of parameter values, though agree to that it may be informative to describe the parameter values in different time periods. To further address the description of those distinct time periods, we have added an additional supplementary table that summarises the parameters during the different time periods in the data from subjects 1, 2, and 3:

Supplementary 4

Days were manually chosen from inspecting the original data. Best fit model parameters from time windows bound by those days were then summarised.

Supplementary Table 1. Mean (SD) fitted parameter values in 1- and 2-nap sleep patterns.

| Sleep patterns | | Infant 1 | Infant 2 | Infant 3 |
| --- | --- | --- | --- | --- |
| 2 naps | | Day 110 - 275 | Day 290 - 510 | Day 110 - 345 |
|  | $\mu$ nM s^-1^ | 8.20 (0.57) | 7.75 (0.51) | 5.86 (0.53) |
|  | $\chi$ h | 32.4 (3.55) | 30.6 (1.99) | 32.0 (5.51) |
|  | $\nu_{vc}$ mV | -3.45 (0.44) | -3.73 (0.63) | -1.62 (0.53) |
|  | $b$ | 0.93 (0.04) | 0.97 (0.02) | 0.989 (0.03) |
| 1 nap | | Day 280 - 548 | Day 520 - 535 | Day 350 - 370 |
|  | $\mu$ nM s^-1^ | 7.80 (0.06) | 7.8 (0) | 5.82 (0.25) |
|  | $\chi$ h | 44.9 (0.56) | 41.0 (5.18) | 36.6 (1.55) |
|  | $\nu_{vc}$ mV | -2.26 (0.05) | -2.55 (0.39) | -1.28 (0.13) |
|  | $b$ | 0.95 (0.004) | 0.93 (0.03) | 0.99 (0.05) |

*“All four datasets appear to converge to individual-specific values of the parameters, ...” How do you define converge? It does not seem to apply to all parameters.*

We have clarified this sentence on page 7:

All four **Infants** appear to **have** individual-specific **best fit** values of the parameter, with the differences in 𝜇 reflecting the individual differences in TSD.

*When fixing some parameters what is the rational for using the particular values?*

We have added extra text to page 10 to explain how we chose the values:

For the remaining subsets with fixed $\mu$, we used $\mu=8.1$ nM s (**grid value of** $\boldsymbol{\mu}$ **that was the best fit for most of the time when Infants 1 and 2 had consolidated, consistent sleep patterns,** cf. Figure 4). We also found that fixing 𝜒 to an adult value ($\boldsymbol{\chi}$ **= 45 h)** tends to increase the fit costs, with those subsets tending to fit worse than those fixing $\nu_{vc}$ and 𝑏 **(**$\boldsymbol{\nu}_{\boldsymbol{vc}}\boldsymbol{=-3}$ **mV as most of best fit** $\boldsymbol{\nu}_{\boldsymbol{vc}}$ **values were between -2 and -4 mV so we selected the middle of that range, and** $\boldsymbol{b=1}$ **given most of the infants best parameter fit involved** $\boldsymbol{b=1}$**)**.

*Fig. 5: also provide the parameter values when you fixed two of them.*

To help clarify this, we have provided more details in the Figure 4 caption on page 11:

Figure **4**: Relative contributions of subsets of parameters at different ages in infancy. a) The minimum cost function value over time for different parameter constraints in Dataset 1. b) The total cost value over the first 200 days of Dataset 1 (seven day windows from midday 5 to 204). c) The total cost value over the last 200 days of Dataset 1 (seven day windows from midday 345 to 544). Subsets of parameters with two fixed parameters have the fixed value from the corresponding single fixed parameter sets (connected by dashed lines in b and c), **that is** $\boldsymbol{\mu=8.1}\mathbf{nM}\mathbf{s}^{\boldsymbol{-1}}\boldsymbol{,\chi=45}\mathbf{h}\boldsymbol{,}\boldsymbol{\nu}_{\boldsymbol{vc}}\boldsymbol{=-3}\mathbf{mV}\boldsymbol{,}$ **and**$\boldsymbol{b=1}$.

*Fig. 5 b,c: how was “Total Cost” determined?*

The figure caption describes the time range of the data used. The total cost equation has been added to the Methods section on page 19:

**With Total Cost normalised to a time period of** $\boldsymbol{n}$ **days given by,**

$$\mathbf{Total Cost}\boldsymbol{=}\frac{\boldsymbol{1}}{\boldsymbol{n}}\sum_{\mathbf{n days}} \mathbf{Cost}\boldsymbol{,}\left( \boldsymbol{14} \right)$$

*Did you run simulations with parameters corresponding to the values of the smoothed parameter curves? Would be very interesting to see the resulting patterns.*

Those smoothed trajectories in Figure 3 are illustrative to guide the reader’s eye rather than formally estimated, so we prefer to not give too much emphasis to them, and now clarify this on page 7.

We repeated the fitting for the other infants. The trajectories of best-fitting parameter combinations for each infant are shown in Figure **3 (smoothed trajectories overlaid as an illustrative aid)**.

*Discussion:*

*“The model could also be used to develop optimised sleep schedules for children, ...” why should sleep schedules be optimized in infants?*

Sleep is important for neurodevelopment and general health. If slight changes to a sleep schedule can improve sleep for infants, this could impact neurodevelopment, mood, cognition, etc. We have added to this point in the discussion on page 14:

The model could also be used to develop optimised sleep schedules for children**, where improvements in sleep could lead to improvements in neurodevelopment and cognition,** similar to work that has been done for adolescence [16, 48].

*Jenni et al. (2005) investigated developmental changes in the homeostatic process during adolescence and found that the buildup of the homeostatic process got slower (longer time constants for buildup) with increasing age whereas the dissipation of sleep pressure (time constant of exponential decline) of remined similar. Further, the distance between the asymptotes (upper and lower) was increasing with age. How would these observations relate to your findings at an earlier age?*

We have added commentary into the Discussion on page 12 as to how our findings link up to Jenni et al. (2005): These findings are consistent with previously proposed theories [27] and consistent with previous modelling work showing that such homeostatic differences drive more rapid sleep/wake cycling in children [37], and in species that sleep polyphasically, similar to human infants [21, 22]. **This result also aligns with that of Jenni et al. (2005) and Skeldon et al. (2016), whereby the rate of accumulation of sleep pressure during wakefulness (**$\boldsymbol{\mu}$**) continues to decrease throughout adolescence from the higher childhood values to the lower adult value.**

*Methods:*

*Implementation: Overall, the description lacks adequate detail.*

*“The model was solved for an initial time of 4 weeks to remove transients caused by initial values, then using the last value of the initial run as the starting values”. Which parameters were used? Does this apply to each point in the grid search?*

*“..., solved for a time period of 35 days to produce a sleep/wake state time series. We calculated sleep characteristic summary measures on the last 14 days of the generated time series.” Same questions as above.*

*Parameter grid search: did you explore all possible parameter combinations or how is simultaneous varying of parameters to be understood?*

We have added more details into the description on page 17 to address the above points. Additionally, yes, we explored all parameter combinations as described in Table 2. The edit now clarifies this point to readers:

The model was solved **for all combinations of parameter values as listed in Table 2,** for an initial time of 4 weeks to remove transients caused by initial values, then using the last value of the initial run as the starting values, solved for a time period of 35 days to produce a sleep/wake state time series.

*Fitting to empirical data: step size for the sliding window? Was an overlap implemented? Different parameter combinations might lead to “optimal” cost values. How do you deal with such a situation.*

The sliding window had an overlap of 6 days (sliding step of 1 day). This has now been clarified in the text on page 19.

To facilitate fitting, for both the data and the model simulations we calculated the probability of being asleep by clock time, using half-hour clock time bins, computed using 7-day sliding windows for averaging (long enough to aggregate data, and shorter than the timescale on which most developmental changes occur) **with 6-day overlap (sliding step of 1 day)**.

As described at the bottom of page 19, ties in cost function value were decided based on closest pacemaker value to 24 h. We have now made this clearer:

Ties in **optimal** cost function value **between parameter combinations** (which could often occur when $\nu_{vc}$=0 resulting in 𝑏 having no effect on sleep patterns) were decided based on the closest pacemaker period to 24 h.

*How does the search relate to Supplementary Fig. 2? This figure is insufficiently described.*

Supplementary Figure 2 presents the distribution of cost function values across all parameter combinations fitting to the 6 time points presented (7-day window centred at days 5, 100, 200, 300, 400, and 500). We have added text describing Supplementary Figure 2.

Supplementary 2

**Supplementary Figure 2 presents the distribution of cost function values (Equation 13) from all parameter combinations considered (Table 2) for the seven-day averaged window of sleep/wake behaviour centred on days 5, 100, 200, 300, 400, and 500 from Infant 1. The high density at the low end of the distribution for Day 5 shows the lack of identifiability in the results, where many parameters can produce sleep/wake behaviour with a similar cost function value. By contrast, for the later days (300, 400, and 500) the lower tails are flat, indicating that only a small number of parameter combinations can reproduce similar sleep/wake patterns.**

*Typo: “Systematically fixing one parameter (cool colours in Figure 6a) or ...” should be Figure 5a*

We thank the reviewer for noting this typo. We have corrected it.
