# Supplementary material for: Mapping the physiological changes in sleep regulation across infancy and young childhood
Source: PLoS Comput Biol. 2024 Oct 21;20(10):e1012541. doi: 10.1371/journal.pcbi.1012541 (PMC11527290; doi:10.1371/journal.pcbi.1012541)
Supplement: Attachment — Submitted filename: PLOS CB Response to Reviewers second 20241002_final.docx [file pcbi.1012541.s004.docx]

Response to Reviewers for "**Mapping the physiological changes in sleep regulation across infancy and young childhood**" for consideration at **PLOS Computational Biology**.

Reviewer comments are in *italics*, response to reviewers are in blue, and manuscript text is in red with edit to texts **bolded red**.

We thank the reviewers for their helpful feedback, and appreciate the opportunity to clarify some final points and improve our manuscript.

**Reviewer 1**

*Reviewer #1: I thank the authors for addressing my comments. I believe the manuscript is suitable for publication in its current form and have no further concerns.*

We thank Reviewer 1 for recommending publication of our work.

**Reviewer 2**

*Reviewer #2: Overall, I’m ok. with the changes and clarifications made. However, one point still needs further clarification, as noted in the comment below.*

We thank Reviewer 2 for their positive appraisal of our work, and have responded to each of their minor points below.

*It's unfortunate that no simulations were presented. While the paper focuses on parameter estimation and interpreting their changes over time, it has not been shown whether the estimated parameters lead to realistic sleep-wake patterns in simulations. Only the probability of being awake or asleep was demonstrated. Therefore, the statement in the discussion, “The model reproduced the characteristic changes in sleep duration, sleep fragmentation, and sleep timing in the first 2 years of life,” is an overstatement and should be removed or revised.*

We have revised the suggested sentence to make clearer that it is the probability of being awake or asleep across time that the model reproduced (Discussion, page 11):

The model reproduced the **observed changes in the probability of being asleep in 30-minute time bins throughout the day in the first 2 years of life, reflecting the characteristic maturation of sleep duration, sleep fragmentation, and sleep timing**.

*Minor: Supplementary Figure 3 a,b: Please use the same dimensions for plotting as in Figure 2 a,b to facilitate visual comparisons.*

We have changed the dimensions of Supplementary Figure 3 to match Figure 2. We have also edited the caption for Supplementary Figure 3 to make the visual comparison clearer.

*Supplementary Figure 3:* Fitted trajectory of sleep maturation for Infant 1, with and without pacemaker period constraint. a) The sleep/wake patterns for each best-fitting parameter combination, **replicated from Figure 2 b for visual comparison**. b) The sleep/wake patterns for each best-fitting parameter combination, fitted without the pacemaker period constraint. c) The trajectories of best-fitting parameter combinations, superimposed with a Loess smoothed line (thick lines) with smoothing parameter $a_{Loess} = 0.2$ that produce the sleep/wake patterns in a), **replicated from Figure 2 c for visual comparison**. d) The trajectories of best-fitting parameter combinations, superimposed with a Loess smoothed line (thick lines) with smoothing parameter $a_{Loess} = 0.2$ that produce the sleep/wake patterns in b).
